# Striving to make the right decision when seeking emergency care: patients’ stories of navigating support alternatives in a digital society, a qualitative study

**DOI:** 10.1186/s12873-026-01505-y

**Published:** 2026-02-25

**Authors:** Annie Axelsson, Ainhoa Goienetxea Uriarte, Joeri van Laere, Martin Gellerstedt, Rajna Knez

**Affiliations:** 1 School of Health Sciences, University of Skövde, Post Box 408, Skövde, SE-541 28 Sweden; 2 Skaraborg Hospital, Lövängsvägen, Skövde, SE-541 42 Sweden; 3 School of Engineering Science, University of Skövde, Post Box 408, Skövde, SE-541 28 Sweden; 4 School of Informatics, University of Skövde, Post Box 408, Skövde, SE-541 28 Sweden; 5 Institute of Neuroscience and Physiology, University of Gothenburg, Gothenburg, Sweden

**Keywords:** Emergency department, Emergency care, Digital health, Healthcare-seeking behaviour, Decision-making, Health literacy

## Abstract

**Background:**

Healthcare systems struggle with an imbalance between the required resources and existing demand. The number of patients seeking emergency care has increased steadily, placing additional strain on emergency departments, which are also challenged by a high volume of non-urgent visits. When patients perceive a need to seek emergency care, the decision is influenced by various factors, including the availability and quality of support and the information they use to make informed decisions. Digital health support encompasses a range of technologies designed to assist at various stages of the healthcare continuum. They are intended to support patients and personnel, as well as effective resource utilization in healthcare. More knowledge is needed to understand the factors involved when patients decide to seek emergency care, and how digital health support can be introduced to benefit both patients and personnel. This study aims to identify and understand important factors that influence patients’ decisions when seeking emergency care, focusing on the information they gather and various sources of support, especially digital health support.

**Methods:**

This qualitative interview study included 31 participants seeking care at an emergency department in western Sweden. The data were analysed through reflexive thematic analysis.

**Results:**

The analysis resulted in four themes: (1) human support needed; (2) going back and forth about making the right decision; (3) digital reassurance; and (4) digital support with hesitation. The participants based their decisions on various forms of information and support. Patients who used digital health support reported mixed experiences, describing both opportunities and barriers. Some patients mentioned expectations for future digital solutions, mainly expressing a desire to preserve human contact.

**Conclusion:**

Patients strive to make the right decision about whether, when, and where to seek care in perceived urgent situations. In this decision-making process, patients use human and digital support for guidance and to facilitate their decision. However, navigating the healthcare system is challenging for patients, and digital health support provides both enabling opportunities and several obstacles. The results of this study indicate that further development of digital health support for patients seeking emergency care would benefit if end users were included in the co-creating process.

**Supplementary information:**

The online version contains supplementary material available at 10.1186/s12873-026-01505-y.

## Background

The number of patients seeking emergency care is increasing steadily in many countries, leading to long waiting times and crowding in emergency departments (EDs) [1]. The number of ED visits in Sweden is approximately 1.8 million visits per year, with an increasing trend [2]. The consequences of an overloaded ED include greater risk for patient safety, treatment delay [3], and negative effects on healthcare professionals’ working environment [4]. In addition, EDs face challenges in recruiting staff and effectively managing available resources, resulting in an imbalance of resources to meet the increasing number of patients [5]. The high number of patients visiting the ED for non-urgent care contributes to ED crowding [6], and one opportunity to ease the burden would be to decrease the proportion of unnecessary visits [7]. The lack of availability and confidence in primary care, a perceived need for more high-quality emergency care, and perceived urgency are some of the reasons for these non-urgent visits [1, 8]. There is no consensus in the literature, and depending on the criteria for assessment, the proportion of non-urgent visits at EDs is estimated to be within 20–40% [9] or 5–90% [10].

It is essential to understand the patients’ pathways before seeking urgent care to reduce the number and proportion of non-urgent visits. When patients perceive a need for urgent care, they face the challenge of whether, when, and where to seek care, resulting in a complex decision-making process [11]. Patients must first assess their healthcare needs, identify an appropriate provider, determine how to reach them, and navigate the healthcare system. A review of the current research has investigated this decision-making process and the factors affecting it [8]. The literature shows that it is not easy for patients to know when and where to seek care [11] and to understand the urgency level of their health status [8]. Furthermore, the decision to seek care is made in a perceived urgent situation by the patient, which may add stress and affect their ability to search for support [8]. In addition, there are predisposing, enabling, and need factors, both individual and contextual, that influence patients’ choice of health service use [12]. A recent study examining patients’ choice of EDs over primary care and other healthcare services revealed that patients’ perceived severity of symptoms primarily affects their decision [13]. Other studies, focused on specific patient groups, such as chronically ill elderly individuals [14] and young adults [15], showing the complexity of the decision-making process. Patients and family members, such as parents with young children, use different types of support to make an informed decision on where to seek care [16–19]. They may turn to informal support from family and friends [16] or seek healthcare professionals via a primary care centre or teleconsulting [14].

There is no doubt that we live in a digital society, and patients’ pathways, when they seek emergency care, most likely involve digital technologies. In this context, the World Health Organization emphasizes that the digitalization of healthcare has the potential to improve health outcomes if it is properly developed and supported [20]. In Sweden, different digital welfare services are offered, such as the Swedish national medical teleconsulting centre (1177), and the country ranks high for digitalization [21].

Digital support involves computer hardware and software that individuals or groups use in organizations to manage information, achieve organizational goals and collect and store data [22]. Digital health is described as different technologies for information sharing, education, and communication that support all stages of healthcare within a spectrum from prevention, diagnosis, and management of health conditions [23]. The combined term “digital health support” is used in this article to refer to digital health technologies that may be supporting and enabling individuals to manage their health. In the patients’ decision-making process, several digital health supports may be relevant, such as searching the internet, making telephone or video calls, using mobile health applications, as well as synchronous or asynchronous chats.

Patients seem to be motivated to use digital alternatives when turning to healthcare [24] and are positive towards using digital health support [25, 26]. Digital health support could also facilitate decision-making when seeking care for acute illnesses [18, 27]. Furthermore, studies show that patients search the internet before visiting EDs to understand unknown symptoms [28, 29]. However, inaccurate information or a lack of knowledge to understand the meaning of the results of the search may also affect the decision-making process [30, 31]. In addition, health literacy affects the decision-making process [32]. Specifically, digital health literacy (i.e. digital competence with regard to health issues) is a factor when analysing the benefits that digital health support may bring when seeking care [33].

Differences in the ability to use digital technologies have an impact on equality in care [34–36]; and without adequate support, digital health initiatives risk widening inequalities in healthcare access for patients [36]. However, digital health support may also reduce inequalities by making patients better informed [37], improving their access to care, and contributing to patient empowerment [37, 38]. Consequently, digital health support has been highlighted as an element in decision-making when seeking care for perceived acute needs [18], offering a supportive, stimulating and interactive role [39].

Digital candidacy (i.e. how patients navigate the complex network of human and digital agents) [36] is also an important dimension that needs to be considered in relation to digital technology and health issues. This navigation process requires patients to have both technological and social competencies, as well as knowledge of the healthcare technologies [36].

Although previous research has highlighted the importance of digital health support, there is a need to further understand its role in patient decision-making when seeking care in the ED for perceived acute symptoms [18, 39], considering both obstacles and possibilities. This study aims to identify and understand important factors that influence patients’ decisions when seeking emergency care, focusing on the information they gather and various sources of support, especially digital health support. The research questions to be examined are as follows: (1) What kind of support formed the basis of patients’ decisions to seek care in the emergency department? and (2) What are the patients’ experiences and expectations of the use of digital health support when they need to seek emergency care?

## Methods

This study was designed as a qualitative interview study [40] conducted at the ED of a regional hospital in western Sweden, where patients with somatic symptoms are treated. This ED receives around 63,000 visits annually and serves patients of all ages.

The participants in this study were patients seeking care at the ED and in the waiting room before triage or being seen by a physician. The study followed the Ethical Review Act (SFS 2003:460), GDPR and Declaration of Helsinki [41]. The interviews were conducted in a potentially stressful context for the participants. Therefore, the interviewer strived to maintain a high level of ethical awareness to respond to any expressions of vulnerability or discomfort from the participants and to avoid any criticism of the decision to seek care in the ED. No assessment was made in this study on whether the patient’s visit to the ED was unnecessary.

The inclusion criteria were patients > 15 years of age and patients < 15 years with an accompanying guardian who were Swedish or English speakers; those who were not critically ill; and those considered well enough by the healthcare staff at the ED to participate in an interview. The participants were chosen by convenience sampling so that participation would not affect their time to treatment. In addition, the recruitment considered variations in gender, age of respondents, days of the week and times of the day when the interviews were conducted. All interviews were conducted by the first author on six different occasions within five months in 2024. The interviewer (AA) asked the patients to participate and informed them verbally and via written information about the purpose of the study and that their participation in the study would not affect their care or time to treatment. Those who agreed to participate were asked to sign a consent form.

Thirty-one persons agreed to participate in the study (16 females and 15 males, two of whom were accompanying guardians of children) three individuals declined participation. The age range of the participants was 17–80 years. Nine were under 40 years old, ten were between 41 - 60 years old, and ten were older than 60 years old (two were not registered by age).

The interviews were based on an interview guide with open-ended questions (Additional file 1). It included questions on the steps taken before they arrived at the ED, whether they had contacted other healthcare providers or consulted another type of support, their use of digital health support to find information about their perceived acute health status, and experiences and expectations of digital health support.

The interviews were conducted when the patient arrived at the ED in a separate room and lasted for 6–15 minutes. The interviews were audio-recorded and transcribed by the first author. The transcribed files were imported into the NVivo [42] analysis programme to structure and sort the data. The data were analysed through reflexive thematic analysis as described by Braun and Clarke with a critical, realist approach [43, 44]. The analysis was conducted primarily by the first author, with regular consultations and consensus among all authors throughout the process. In reflexive thematic analysis, the researcher’s subjectivity is considered a resource when conducting interviews and analysing data [45]. The first author’s experiences working as a nurse in an ED and the clinical and/or research experience of the other authors in this area were considered a resource during the interviews and when interpreting the data through an inductive and latent approach. The analysis process in a reflexive thematic analysis aims to construct patterns of meaning as outputs from the data in six defined phases [43, 44]. The way these phases were carried out in this analysis is outlined in Table 1.

**Table 1:** Description of the different phases of the data analysis process using reflective thematic analysis

The six phases of reflective thematic analysis^43^	Description of the process
1. Familiarize yourself with the dataset	Familiarization was achieved by listening to, transcribing, and reading the interviews.
2. Coding	The initial codes were developed systematically by labelling and coding the data that answered the aim. This process included reading the data and codes several times. These codes were developed and changed iteratively during this process. Notes were made in a reflective journal.
3. Generating initial themes	The initial themes were developed by interpreting the aggregated meaning and meaningfulness in the data.
4. Developing and reviewing themes	Codes and data were read and compared to find patterns of sentences that could develop into themes or subthemes; eight themes were developed initially.
5. Refining, defining and naming themes	The initial eight themes and all codes were discussed thoroughly and refined by all the authors together several times before the final themes were defined.
6. Writing the report of the analysis	The results of the analysis are reported in this article.

## Results

The analysis of the interviews resulted in four themes: (1) human support needed, (2) going back and forth about making the right decision, (3) digital reassurance, and (4) digital support with hesitation.

### Human support needed

This theme encompasses the fact that participants turned to relatives and/or healthcare professionals for consultation and support in their decision-making process. According to the results from the interviews, getting support from another person is important when thinking and interpreting healthcare-related issues. The participants expressed that it was reassuring not to be alone in the decision about how to proceed when seeking emergency care. Some detailed reflections and citations from the interviews are presented below.

Assistance from relatives or friends can suggest to patients that there is no urgency and make them confident about not seeking care.

If it’s not threatening, you don’t need to take up their [healthcare personnel's] time.

Nevertheless, support from relatives sometimes also helped them speed up the process of seeking care at the ED if the relative believed that there was an immediate need for care.

No, I never seek care; I wait as long as possible, and now it was my wife who said that I should come to the ED, so I did.

Some participants discussed the symptoms with people they trusted or had better medical knowledge or experience, hoping that they could support them in making the right decision on whether and where to seek care.

Oh, I think it is good to be able to call a friend who is wise about the body and disease. The person I call is good at that.

Turning to the nearby primary care centre or calling the Swedish national medical teleconsulting centre (1177) was an established way to obtain assistance and acquire confident information among participants. This, in line with turning to their regular specialist clinic, may be the first step for participants to receive professional support. This kind of support provided them with someone to discuss things with, to sort things out at home, and to guide them further. It gave them peace of mind about what to do.

You can do quite a lot at home before you, well … so you get someone to direct you on what to do with the problem or injury you have. In addition, I think that is pretty good … to get someone to discuss it with, before you decide what to do.

They often received support they could rely on from relatives and healthcare personnel. However, some did not perceive that they received the help they needed from the healthcare professionals, resulting in the decision to visit the ED.

… at the health centre in [Name], oh, you don’t get any contact, and you don’t get any help.

Several participants felt that they did not decide to visit the ED themselves and that the final decision to visit the ED was taken by someone else.

I called a friend … she said that … ‘well, that’s the emergency department’. But I said no. But she said to at least call 1177 and talk to them, and I did, and they said the same thing. Go straight in!

Participants who received referrals or advice from healthcare professionals or relatives, or who relied on others’ decisions, expressed mixed emotions. Some felt completely comfortable, whereas others experienced doubts about whether they truly needed to seek treatment in the ED at all. However, based on the recommendations or referrals, they proceeded, feeling reassured that it was the right decision. Without that firm guidance, they might have chosen differently, perhaps not seeking treatment at all or consulting another healthcare provider.

I had wanted to wait until Monday to seek care … but now I was advised against that.

Participants followed the advice almost like a referral. They trusted the professionals and felt that they were receiving the right guidance.

Hm, well, I first called the healthcare centre and asked what they thought. Oh, then they said that it would be quickest to go directly here.

### Going back and forth about making the right decision

This theme describes that participants’ decisions are shaped by information and support in different ways. According to the results from the interviews, the participants wanted to do the right thing, considering individual and contextual factors such as their perceived needs, their knowledge and previous experience of the most suitable places to seek care, as well as the availability of care providers.

Participants’ decisions on where to seek care often followed a winding path, sometimes unfolding over a long period, sometimes quickly, before ending up at the ED. Seeking care at the ED is not the first step taken by participants.

But there is an inner barrier. For me, there is anyway. No, I’m not going to seek care there, it’s not that serious, it’s for the extremely sick, I think.

Decisions were often carefully considered, drawing on previous experiences or information provided by a clinician, even if it was from several years ago, to choose the most appropriate provider.

The doctor said [some years ago], that ‘if you have pain again, we will send you to a surgeon right away; there is no medicine, only surgery’.

Participants’ decisions were sometimes made based on the information and support they received when they turned to healthcare providers.

Oh, I was going to do some different exercises, but this is more urgent, I think, so I called in here today.

Some felt that turning to a primary care centre was irrelevant if they believed that they needed a deeper examination, such as an X-ray or a more specialized evaluation. They believed that the ED had the right resources in these cases. Some patients felt that the ED was the only place where their symptoms would be thoroughly examined and they would be truly heard, offering a sense of reassurance. Sometimes, there was also frustration and despair among participants, caused by a lack of adequate help in other healthcare instances.

I do not feel like I used to. I have visited the primary care centre at home twice, and I do not feel that I am getting any help. I cannot bear this anymore.

The reasons why participants felt it was urgent to visit the ED straight away without consulting other healthcare providers varied. It might have been pain or other medical symptoms that escalated quickly. Sometimes the increased symptoms made them very worried, driving the decision.

But then, anyway, this morning I called my daughter and said that now I can’t stand the pain anymore, so then I made the decision.

Nevertheless, there were some non-medical reasons, such as their personal schedule or being at the hospital for other errands.

Since I’ve had [the symptom] for a long time, I thought that when I’m here at the hospital, I could go to the emergency department.

Some participants also considered contacting professionals before seeking help at the ED, but chose not to do so, because doing so was more complicated than just going to the ED. Likewise, if they failed when trying to reach other providers.

Yes, I’ve tried it, but I’ve probably phoned 6 or 7 times this morning, but you can’t get through. It just says that: ‘there are so many people calling now, so you have to call in a while’.

After gathering information, the uncertainty of whether the symptoms were indications of a severe disease was sometimes difficult to manage on their own because it created too much anxiety. Participants even described that those questions sometimes arose after their last contact with a healthcare professional, initiated by the professional, and leading to worry and feelings of vulnerability.

But sometimes you think they should ask. As the doctor who called me, [the doctor] should have asked, ‘Are you home by yourself?’ But [the doctor] didn’t! [The doctor] just gave it to me! And then it was the weekend, the whole weekend! Ugh, it wasn’t good. I really feel so anxious.

Almost all participants felt that they were in the right place when being interviewed. Participants who had made their own decisions and those who had been guided felt confident that the ED was the right place to be. However, some felt that their issues belonged elsewhere, despite being referred to the ED by other healthcare providers, but they accepted the situation.

But it was difficult because an emergency for me means you’re dying, kind of … I’m not dying. I don’t think so anyway. But I sense that I can’t feel like this anymore. So, then it had to be like this.

### Digital reassurance

This theme describes participants’ experiences of digital health solutions to support them in different ways. Digital health support, such as the internet, mobile health applications for information, online visits, or telephone calls to healthcare professionals, relatives, and friends, provided participants with information to establish the basis of the decision to seek care. Sources of digital health support in the form of seeking information on the internet were commonly used and experienced by nearly every participant.

According to participants, online information provided support, was reassuring, and made them feel secure. The informants may be comforted and feel calmer about the situation. It may provide the reassurance they need, to know if they should seek care straight away or if they should wait.

At first, I thought, God, we will probably have to go by ambulance. However, then, when I had calmed down, I started googling. And then I felt calmer and understood that we could stay at home and wait.

Online information made the participants better informed and feel better prepared when meeting the ED personnel or facing the necessary examinations or treatments. Concerns about what lay ahead were mitigated by written materials and online videos.

I’ve watched some videos on YouTube and such. It was good information about surgery. I’ve checked some and read reports and so on, and it’s not dangerous with surgery.

To trust online searches, participants used their own strategies, such as searching on pages they perceived as credible, pages they recognized or knew were reliable sources.

I search a lot online and try to find information there on credible pages.

However, there are also strategies in which, after gathering information, an active decision to trust the sources is required.

There are a lot of things, everything from fantastic information to pure rubbish. You have to sort, and then you have to check the source and decide whether to trust this information or not.

Digital health support also has a role when contacting healthcare providers. A few participants describe the benefits of communicating asynchronously with healthcare professionals through a digital platform.

You get a written answer at least and, even if you can’t take it in at the time, you can read it again and so on.

Furthermore, medical consulting through telephone calls was an important digital health support to participants and was considered to be easily accessible.

I prefer to call …. yes, that’s good and I get an answer right away. Instead of sitting and writing.

However, some participants preferred video calls with a healthcare provider over consultation by phone. The combination of traditional healthcare and digital health support was considered a way to retain human interaction and, at the same time, provide benefit from digital support. For instance, the patient had the opportunity to see the healthcare provider through video calls instead of making a telephone call.

It is good to have a video link there, because it then becomes more personal. The meeting is important, for me and for the doctor. [The doctor] should be able to make a correct evaluation.

Another benefit of video calls mentioned by the participants was the possibility of accessing healthcare without leaving home or the workplace.

You don’t have to travel for counselling because it takes time, and we live in the countryside.

Digital health support in the form of specialized digital health online applications was also experienced as easy and convenient. It was a way of getting both written and video call support and saved both parties time and travel. Some parents of ill children reported previous experience of receiving the necessary assistance peacefully at home, eliminating the need to travel.

Very smooth. My child had [the symptom], and we got help quickly and easily, and it turned out well. It was smooth.

### Digital support with hesitation

This theme involves the challenges experienced by participants when using digital health support and their expectations of this type of support. Digital health support was described as supporting and facilitating, but at the same time challenging, with obstacles and perceived risks.

The functionality of digital health support can sometimes complicate urgent situations. For example, health records from different providers may fail to exchange information or images, or patients may struggle to use a mobile device camera when injured.

I was at home by myself, it was bloody and there were blurry pictures, and I had to send them again, and finally the healthcare provider on the phone call asked me to put my [hand] on the table, then I only had one hand to use the mobile phone.

Knowledge about digital health support in acute situations, varied among the participants. Some viewed it as a natural option to utilize in searching for healthcare, whereas others were unaware of alternatives to traditional in-person visits.

It works quite well actually, but it’s probably because you have gotten used to the digital world. In the digital society today, you have been forced to use such services; it has become quite natural that you do so.

Experience of mobile health applications offered within the public healthcare sector was limited. Solutions developed by private healthcare providers were also perceived as a financial threat to local primary care centres, due to the structure of Sweden’s healthcare funding and allowance system and were therefore not regarded as a viable alternative by some patients.

Yeh, sure, a private app, but then it will be a burden on my primary care centre and that I truly will avoid.

Difficulty recognizing relevant situations and symptoms when using online applications was a clear challenge experienced by the participants. Both users and non-users of these types of applications reported this and indicated a need for explicit instructions on accessing suitable tools.

There are digital ones, but I don’t know, I think it’s difficult with these tools because I don’t really know what symptoms to look for then. If you have a sore throat or something?

Even though participants found relevant information online, encountering serious interpretations of their symptoms often increased stress, especially when they struggled to relate it to their own circumstances.

You can try to find some things but if you keep on googling too much, I think you will get sick of everything in the end. It creates disease instead.

Some participants hesitated to use digital health support for emergencies because the situation was perceived as serious.

I just think, with this urgency … if it is urgent, it feels weird that you should sign in and type a message. If you understand what I mean.

Even though different digital health solutions were viewed as useful and a good complement to regular visits, digital health support sometimes created a feeling of uncertainty and was not considered trustworthy. The participants expressed feelings that artificial intelligence (AI) in digital health support was unreliable and a threat, even a threat to mankind.

It mustn’t be too much technology, not too much. Balanced is good, so that it is not an AI doctor sitting there and assessing. I don’t think that’s good. Not good for humanity.

Perceived challenges with video calls created doubts among some participants. Explaining their symptoms to healthcare professionals without a physical meeting was perceived as more difficult by participants. They were concerned about losing human interaction, and questions arose about the quality of remote assessment.

It feels like you need to have that human contact.

## Discussion

This study aimed to explore factors influencing patients’ decisions to seek urgent healthcare, as well as their experiences of and expectations from digital health support. The findings indicate that patients attempt to make the right choice when they perceive the need to seek medical care. For some, this intention is shaped primarily by their experiences at the time, whereas for others, it reflects a deliberate effort to reach the appropriate care level using human and digital support.

### Patients’ journeys towards the ED with human and digital support along the pathways

The results of this study indicate that patients strive to make the right decision when seeking urgent healthcare, aiming to ensure that their choices align with their own needs and the expectations of the healthcare system. However, this consistency is not easily achieved, given the high number of unnecessary visits to EDs [9, 10] and the misalignment between how the healthcare system and patients define what constitutes a necessary visit to the ED [46].

**Fig. 1:**
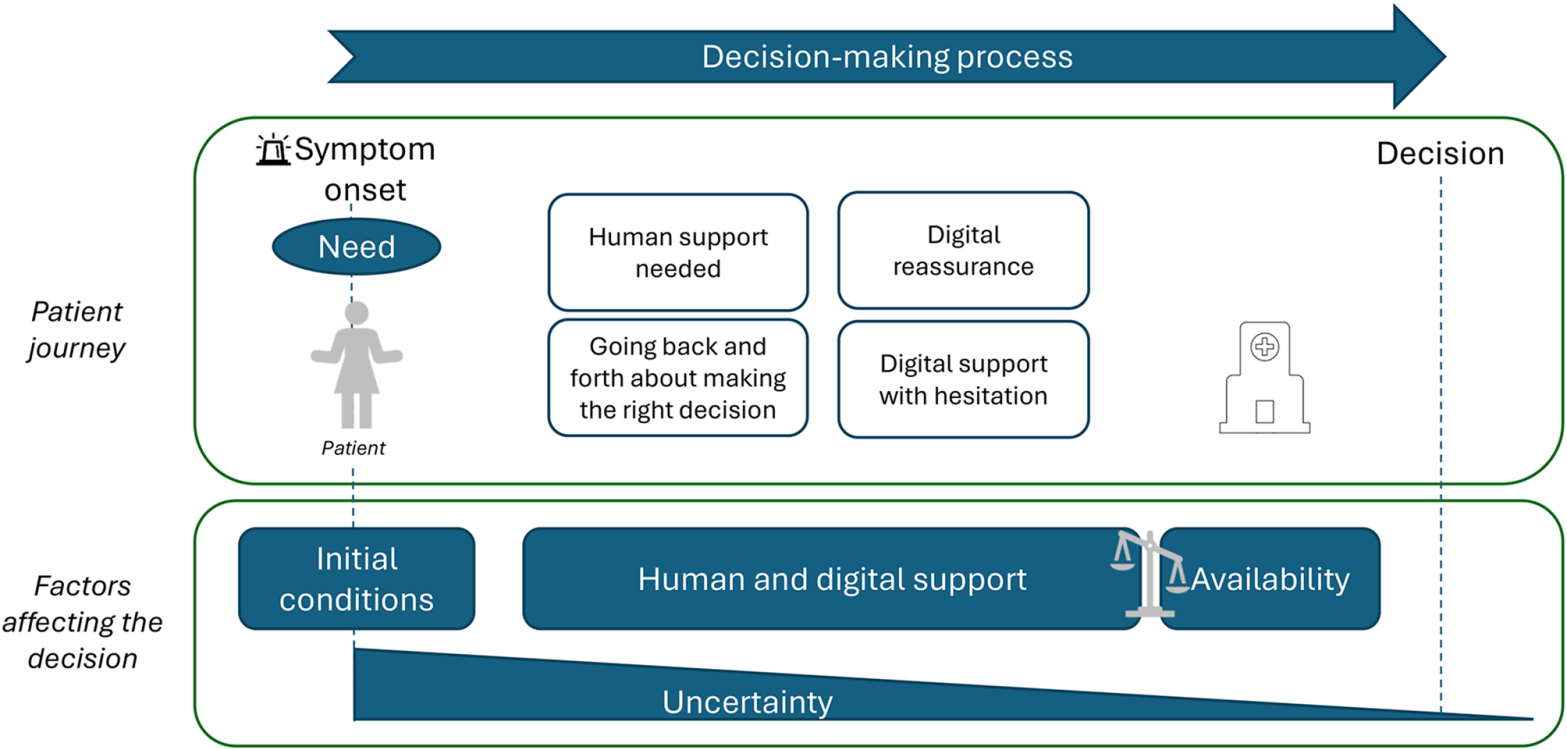
The decision-making process in the patient’s journey to seek care

The patient’s journey towards the ED can vary in length and is not always straightforward. This twisted pathway is understandable because the decision to seek emergency care is a complex process [1, 5, 8, 14–16, 19, 47]. The journey begins when the patient experiences symptoms and perceives a need for medical help, possibly urgently (Fig. 1). The patient’s initial conditions, in terms of their previous experiences of medical care, their biopsychosocial and economic circumstances, as well as (digital) health literacy, can shape their initial thoughts and actions about what to do. These aspects are also presented by other authors as predisposing factors when seeking care [12]. Previous research has also highlighted that the digital dimension adds further complexity to the decision-making process when seeking care, a concept understood as digital candidacy [36].

The onset of symptoms that triggered the patient’s decision-making process may be due to new or previously known symptoms. The need for care may be both a perceived and an actual medical need [12], and may be a complex issue from the patient’s point of view. Patients need to interpret their symptoms and evaluate information from various sources of support, including digital health support. They navigate through a complex socio-technical network of both human and digital agents towards their decision [36]. Furthermore, they need to find their way within the healthcare system.

As indicated in our study, to make informed decisions, patients try to gather information and support in various ways, by turning to digital and human support along their journey (Fig. 1). Advice from relatives, friends and/or consultation with healthcare professionals, along with their earlier experience, as well as information they may have collected digitally, helped form patients’ decisions. The findings in this study also suggest that the use of digital health support is embedded in many patients’ decision-making processes (Fig. 1), or at the very least, patients have encountered and used such support in similar situations. Furthermore, the lack of availability in primary care was pointed out in this study by some participants when describing how they failed to get help or even make contact in some cases. This is in line with earlier studies as a reason why patients turn to an ED [1]. Thus, availability in the different areas of the healthcare system is also an important factor affecting patients’ decisions (Fig. 1).

Participants in this study had experience using different modes of digital health support such as mobile health applications, video calls, and digital platforms for synchronous and asynchronous communication with healthcare providers. However, phone calls to healthcare professionals, relatives or friends and searching the internet were the main methods of digital health support. In the context of patients’ decision-making, the internet plays a role regarding general health issues [39]. This study as well as a few earlier research studies [31], suggest that internet information may facilitate decision-making in perceived urgent situations. Moreover, a combination of digital and human support is considered to facilitate increased learning and a deeper understanding of patients’ health and symptoms; for example, when patients validate digital information with healthcare professionals to understand it fully [39].

The activities that participants in our study performed before entering an ED may reflect patients’ response to their own underlying uncertainty about whether, when and where to seek emergency medical care. Thus, turning to human and digital support might be understood as an attempt to diminish these accompanying concerns and uncertainty, leading patients to an appropriate decision. In this study, participants felt that they were in the right place, regardless of the reason or whether they had been referred or made their own decision to visit the ED. At the same time, many participants felt they should have been able to access help elsewhere, reflecting persistent uncertainty even after visiting the ED (Fig. 1).

The patients’ expression of being in the right place raises the question: was it right for the healthcare system or the patient? Considering the increasing importance of the concept of person-centred care, where the person’s views about their life situation and condition are always at the centre [48], there seems to be a contradiction in placing responsibility on patients for not seeking care at the right level. Debating whether patients seek care in the right or wrong place may be questioned because the definition of what is right diverges [46]. This study reinforces earlier suggestions posed in the literature on whether patients should have the knowledge required to make the right decision when seeking care [14]. Thus, efforts should focus on strengthening (digital) health literacy and empowering patients to actively seek and critically appraise health information, thereby enabling them to make well-informed and more appropriate decisions.

### Patients’ experience and expectations as a foundation for advanced digital health support

This study captures some of the opportunities and challenges of digital health support solutions, based on participants’ experience and expectations.

Digital health support appears to be promising, but some patients are hesitant. The results from this study indicate that being able to search for information on the internet can create confidence before a visit, enhance patients’ sense of security, and empower them to wait or manage their symptoms on their own. However, this study also implies that reading information on the internet can affect individuals negatively, which is in line with previous research [28]. The heterogeneity in how individuals are affected by health information, together with differences in their levels of health literacy, are important considerations when creating online health information [32].

This study indicates that participants have mixed feelings about receiving support digitally. Although they recognize its value as an alternative in certain contexts, they also express hesitation about using asynchronous chat during perceived acute situations, in contrast to its convenient use for non-urgent communication with healthcare professionals. Asynchronous communication can complicate contact with healthcare providers and may increase the patients’ uncertainty about how to seek healthcare [36]. This study also indicates that patients are afraid to lose the human interaction with healthcare personnel in digital communications. This is a concern echoed in previous research showing that replacing the warmth of an in-person interaction with messages and screens can create a sense of dehumanization [36]. Our results also underline patients’ wishes for a combination of digital and in-person support. For instance, video calls were sometimes described as enhancing interpersonal contact compared with a telephone call when interacting with healthcare personnel. Therefore, it is reasonable to believe that digital health support could play a greater role in guiding patients if it is balanced with in-person support.

Further development of digital health support technologies may help guide patients in the right way and decrease their concerns. Digital health support may facilitate patient empowerment and enable patients to meet their healthcare needs [37, 38]. This study also indicates that patients find it difficult to know when, how, and which digital health support to use in emergencies. This implies that these should be designed to reach patients through appropriate channels at the right time. The right time may be before symptoms arise, but people may not familiarize themselves with their illnesses or health issues in advance, and it might be a challenge to find information in stressful situations. Guiding patients to a suitable digital health support in the decision-making process may increase their ability to make well-informed decisions and facilitate the use of digital health support at appropriate times.

Including the general public in the development of digital health support may be a viable approach to increase access to that kind of support for acute situations. As suggested in an earlier study, there is a need to develop more interactive systems that give patients individualized and quicker support [36]. When patients actively participate in the design of healthcare services and healthcare information materials, it better meets their needs and becomes more accessible and easier for patients to navigate [38]. A recent study shows that several digital health support technologies are designed in a way that benefits patients with higher levels of digital or health literacy, or those whose needs align closely with standard or typical care scenarios, potentially reinforcing inequalities in healthcare [36].

### Implications for future research

Future research should investigate how availability and equality in healthcare may be affected by the use of digital health support, particularly in relation to health literacy and digital health literacy. The risk of being denied access to healthcare due to lower digital literacy, as when needing to describe oneself as patient in digital agents [36], should be considered. As noted in this study, video clips from the internet were used in the patients’ decision-making process, reassuring the patient before seeking healthcare. Although this may play a role, it has received limited attention in research. Overall, how to enable patients to make an informed decision about their health by educating people through the use of social digital technologies has not been fully investigated [23] and needs to be incorporated in future studies on the topic. Furthermore, how people integrate digital technologies such as internet video clips, social media, mobile apps, and gaming devices into everyday life and what role these technologies play in health domains need to be further investigated [23]. In addition, quantitative studies of the factors influencing initial conditions, the type of support needed from various digital and human sources, as well as studies that traces every step in time in the patients’ decision-making process when perceiving a need for urgent care, may be of future interest. On the other hand, beyond analysing patients who visit the ED, it is important to investigate the broader population’s decision-making habits regarding whether to seek care at the ED, including those who ultimately choose not to do so. Furthermore, future research should explore the role of digital health support linked to the outcome of visits to EDs and whether digital health support reduces avoidable or unnecessary ED visits.

### Strengths and limitations

The main strength of this study is that it includes patients’ own stories of their decision-making process in an emergency situation. This study also offers a unique contribution by capturing patients’ perspectives on the role of digital health support when seeking care at the ED. However, the study has several limitations. Only patients who decided to visit the ED were included in this study. The inclusion of those who decided to stay at home or receive care from other healthcare providers could capture additional experiences and lead to additional conclusions. It is difficult to trace patients’ steps before arriving at the ED [49], and this study has probably not captured all the details of the patients’ journey before they entered the ED, which is a limitation. In addition, we wanted to capture patients’ thoughts about their decision without being influenced by the outcome of the visit to the ED; therefore, the interviews were performed before triage and designed as short interviews to avoid interfering with their care process. Thus, these short interviews may have limited the patients’ reflections on their answers. Another option could have been to interview the patients after they were attended by the ED personnel; in this way, the interviews could have included more questions as the time pressure decreased. This study may have had different results in another country where the healthcare system and the digital infrastructure are different.

## Conclusions and implications

This study illustrates patients’ journeys when navigating the healthcare system for a perceived need for urgent care and in the broader context of the digital society. The results from the interviews indicate that patients strive to make the right decision and draw on various forms of human and digital support when making care-seeking decisions. However, navigating the healthcare system can be challenging for patients, as shown by this study. The experiences and expectations identified can inform the design and implementation of digital health support initiatives aimed at optimizing patients’ navigation of the healthcare system.

Involving potential users and targeted patient groups in the process of co-designing future digital health support technologies may better support patients’ decision-making, guiding them in a timely manner to the appropriate level of care.

## Electronic supplementary material

Below is the link to the electronic supplementary material.

Supplementary material 1

## Data Availability

The raw data generated and analysed in this study are not publicly available due to ethical restrictions.
