# Supplementary material for: Striving to make the right decision when seeking emergency care: patients’ stories of navigating support alternatives in a digital society, a qualitative study
Source: BMC Emerg Med. 2026 Feb 25;26:68. doi: 10.1186/s12873-026-01505-y (PMC12955183; doi:10.1186/s12873-026-01505-y)
Supplement: Supplementary file 1 — Supplementary material 1 [file 12873_2026_1505_MOESM1_ESM.docx]

**Appendix 1. Interview guide**

***The way in***

Could you describe what influenced you to determine that it was essential to come here (to the Emergency Department) today?

What was the basis for that assessment/decision to come here?

Are the symptoms something new or something you have had trouble with for a long time? Sought care for before?

Have you enlisted the help of another person?

***Support and information***

Have you contacted any other healthcare provider today or recently about your current problems?

If YES, what did they say?

If NO, have you considered contacting someone else?

If YES What stopped you in that case?

Can you describe if you have tried to obtain information about your current problems in any other way?

In what way did that information support you or in what way did it not support you?

***Experiences and expectations***

Do you know of any digital tool that can provide support in relation with acute problems, other problems?

Have you used any digital tool in connection with acute ailments, other ailments?

- Which one(s)?
- What did you think of it?

Describe how you would like to be able to get support and help in choosing where to seek care when you have acute problems.

What do you think about the possibility of digital support before an emergency visit?

Can you describe how you would like to get digital support before emergency visits?

***Examples of follow-up questions***

What do you mean?

Can you tell me more?

In what way was it a support for you?

Can you give an example?

How did you feel about it?

***Background questions***

Age

Sex

Level of education

Occupation

What type of accommodation do you live in

Civil status

Children (approx. age)

Do you have a mobile phone that you use for more than calling, texting, or emailing?

Would you say that you have a good experience using digital solutions such as mobile phones or computers?

Do you get help from someone to take care of digital matters?
